# Noise Suppression on the Tunable Laser for Precise Cavity Length Displacement Measurement

**DOI:** 10.3390/s16091428

**Published:** 2016-09-06

**Authors:** Radek Šmíd, Martin Čížek, Břetislav Mikel, Jan Hrabina, Josef Lazar, Ondřej Číp

**Affiliations:** Institute of Scientific Instruments of Academy of Sciences of Czech Republic, v.v.i., Brno 61264, Czech Republic; cizek@isibrno.cz (M.Č.); mikel@isibrno.cz (B.M.); shane@isibrno.cz (J.H.); joe@isibrno.cz (J.L.); ocip@isibrno.cz (O.Č.)

**Keywords:** Fabry-Perot cavity, unbalance Michelson interferometer, noise suppression, heterodyne interferometry, displacement measurement

## Abstract

The absolute distance between the mirrors of a Fabry-Perot cavity with a spacer from an ultra low expansion material was measured by an ultra wide tunable laser diode. The DFB laser diode working at 1542 nm with 1.5 MHz linewidth and 2 nm tuning range has been suppressed with an unbalanced heterodyne fiber interferometer. The frequency noise of laser has been suppressed by 40 dB across the Fourier frequency range 30–300 Hz and by 20 dB up to 4 kHz and the linewidth of the laser below 300 kHz. The relative resolution of the measurement was $10^{-9}$ that corresponds to 0.3 nm (sub-nm) for 0.178 m long cavity with ability of displacement measurement of 0.5 mm.

## 1. Introduction

Measurement of the length of etalons and measurement of the length of passive Fabry-Perot cavities [1,2,3] or their displacement [4,5] is limited by vibration of mirrors, thermal fluctuations, speed of lock-loops and by the noise and the linewidth of the laser source. The linewidth of the laser should be at least roughly around the linewidth of the passive cavity modes to lock the laser to the cavity by the derivative technique [6,7] or the Pound-Drever-Hall technique [8]. The typical external laser cavity has plano-concave or confocal configuration with a mirror distance from 10 to 500 mm. The finesse of the cavity typically achieve from 100 to 1000 s and in clock laser stabilization cavities up to 100000 s. The linewidth of the typical cavity then reaches between kHz and MHz with pm to nm uncertainty in the cavity length. The measurement of displacement of the Fabry-Perot cavities thus could be made by a tunable laser with sub-kHz to sub-MHz linewidth with an optical reference laser with better or similar linewidth [9,10]. The He-Ne lasers can achieve relative stability of $10^{-13}$ [11,12,13] but the PZT tuning range is limited only to 1 GHz. Typical diode lasers have larger tuning range but the linewidth of 30 MHz that is suitable only for cavities with very low finesse. The Distributed FeedBack lasers (DFB) [14] can achieve roughly 1 MHz linewidth corresponding to $10^{-8}$ relative uncertainty of measurement and 1 nm uncertainty for 100 mm long cavity but offer wide tuning range of nms or hundreds of GHz. Better linewidth can be achieved for external cavity laser (ECL) diodes based on DFB lasers and gratings [15,16] but these laser systems may be quite complex and sensitive to alignment and can introduce power losses. The state-of-the-art ECLs include planar waveguides with fiber Bragg grating and reach linewidths under 3 kHz [17] but the tuning range is only 20 pm or 5 GHz. Further improvement of relative uncertainty of the displacement measurement can be achieved by laser frequency noise suppression. Frequency noise suppression can be achieved by locking and stabilization of the laser to an absorption cell [9,18] or to an ultrastable cavity from very low expansion material such as ULE [19]. These methods have limited tuning range of treated lasers. Laser phase noise suppression was already achieved by the methods based on unbalanced fiber interferometer [20,21]. We have already presented the set-up for the laser noise suppression of planar waveguide external cavity laser module [22] whereas in this work we present the phase noise suppression of a DFB laser diode with wide tuning range to monitor the displacement of the Fabry-Perot cavity with sub-nm uncertainty. We have also presented that the continuous high accuracy displacement measurement with one single mode DFB laser is important in monitoring of the low expansion materials [4] and unlike the displacement measurements for calculable capacitor [23] we are able to track the cavity displacement over the whole tuning range of the laser. This paper includes the measurement set-up for cavity length displacement and new results including the measurement of beat frequency to a stable optical frequency comb.

## 2. Theory of Method

### 2.1. Principle of Displacement Measurement

The displacement measurement of the Fabry-Perot cavity (FPC) is based on the monitoring of the beat frequency between the single mode narrow-linewidth laser locked to the cavity and the closest optical frequency comb tooth.

For components of optical frequency comb:

(1) $$f_{i}=f_{0}+i\cdot f_{r}$$

with $i\approx 10^{6}$ number of comb tooth, where $f_{r}$ and $f_{0}$ are repetition and offset frequency. Both frequencies and the optical frequency of the comb are stabilized based on f-2f stabilization technique [24] and represents the ruler over wide optical range. The optical frequency transmitted through the FPC is defined as:

(2) $$\nu =m\cdot \Delta \nu_{\mathrm{FSR}}$$

where *m* is integer number and $\Delta \nu_{\mathrm{FSR}}$ is the free spectral range (FSR) of the cavity [5]:

(3) $$\Delta \nu_{\mathrm{FSR}}=\frac{c}{2\cdot nL_{\mathrm{cav}}}{\left(1+\frac{\alpha }{2\pi }\cdot \frac{c}{nL_{\mathrm{cav}}}\right)}^{-1}$$

where $L_{\mathrm{cav}}$ is the vacuum cavity length, *n* is the refractive index of air between the mirrors of the cavity, *c* is the vacuum speed of the light and *α* is the coefficient representing dispersion on the cavity mirrors. Following the conditions in [5] we can write for measurement of the displacement of the cavity within the small range of wavelengths α=0. For an optical frequency of single mode laser:

(4) $$\nu =f_{i}\pm \delta f=f_{0}+i\cdot f_{r}\pm \delta f$$

where ±δf is the beat frequency between the single mode laser and the closest optical frequency comb tooth. In confocal geometry one can write using Equations (1)–(4) for the length of the cavity including the beat frequency from the single mode laser and the comb:

(5) $$L_{\mathrm{cav}}=\frac{mc}{4n(f_{0}+i\cdot f_{r}\pm \delta f)}$$

for displacement $\Delta L_{\mathrm{cav}}=L_{\mathrm{cav}2}-L_{\mathrm{cav}1}$

(6) $$L_{\mathrm{cav}}+\Delta L_{\mathrm{cav}}=\frac{\left(m+\Delta m\right)c}{4n\left[f_{0}+\left(i+\Delta i\right)\cdot f_{r}\pm \Delta \delta f\right]}$$

hence the displacement of the cavity is related to the change of the cavity modes $\Delta m=m_{2}-m_{1}$, the number of swept comb teeth $\Delta i=i_{2}-i_{1}$ and change in the beat frequency between the stabilized optical frequency comb and the tunable single mode laser $\Delta \delta f=\delta f_{2}-\delta f_{1}$.

### 2.2. Principle of Noise Suppression

The method of frequency noise suppression and narrowing of laser linewidth in the fiber interferometer is based on the fact that the laser beam is split into two arms and produces two coherent sources. Two arms of interferometer are not of the same length and the signals from two beams mix on a fiber splitter and contain the information about the initial beam and the delayed beam. The system transfer function for Fourier frequency *f* is defined as:

(7) $$T\left(f\right)=\frac{1-e^{-2\pi j\tau_{0}f}}{jf}$$

where *j* is the imaginary part and $\tau_{0}$ is the delay of the laser beams between two interferometric arms. The power spectral density of frequency fluctuations based on the Wiener-Khintchin theorem for stationary distributed signal depends on the autocorrelation signal $R_{\nu }\left(\tau \right)$ in the time interval τ∈〈−∞,∞〉 between the short and long interference arm:

(8) $$S_{\nu }\left(f\right)=\int_{-\infty }^{\infty }R_{\nu }\left(\tau \right)\exp\left(-2\pi f\tau \right)d\tau$$

There are two types of interferometric detection methods:

- **homodyne interferometric detection.** The laser beam splits into two arms and the beam that comes from the longer arm is directly mixed with the beam coming from the shorter arm. The detected signal represents phase noise of the laser and the signal is set around DC. One disadvantage of such a method is that the flicker noise overlaps the detected signal. The bandwidth of frequency noise is limited by the equation [20]:
  (9) $$cos(2\pi \nu_{0}L_{0}/c)=0$$
  where $\nu_{0}$ is the laser frequency, $L_{0}$ is the length difference between two fiber arms of the interferometer. In other words only certain discrete frequencies can be transmitted limited by the actual length of delay fiber:
  (10) $$\nu_{0}=\left(1/2+k\right)\frac{c}{2L_{0}}$$
- **heterodyne interferometric detection.** The laser beam splits into two arms and the optical frequency of the beam in one arm is frequency shifted by $\Delta f_{\mathrm{AOM}}$ on an acousto-optic modulator (AOM) driven by the harmonic RF signal.

We have chosen a heterodyne approach that means that we added an additional frequency shift $\phi_{\mathrm{RF}}$ to the longer arm. Then for the condition in Equation (10):

(11) $$cos(2\pi \nu_{0}L_{0}/c-\phi_{\mathrm{RF}}\left(\tau \right))=0$$

where $\phi_{\mathrm{RF}}\left(\tau_{0}\right)$ is the phase difference between the modulation and demodulation signal. The frequency is the sum of Equation (10) and $\phi_{\mathrm{RF}}$ and thus it can be more easily tuned [20]. There are two common types of interferometer:

- **Mach-Zehnder unbalanced interferometer** where one arm is represented by the fiber spool with active length $L_{1}$. The total delay of the interferometer is $\tau_{0}=\frac{n\cdot L_{0}}{c}$. That is for a typical optical fiber at 1550 nm (SMF-28 [25]) with a fused silica core and refractive index of $n_{g}=1.4682$, thus the delay $\tau_{0}$ ranges from 5.4 ns for 1 m long fiber to 5.4μs for 1 km long arm.
- **Michelson unbalanced interferometer** where one arm is represented by the fiber spool and both arms are ended by the Faraday mirrors. The active length $L_{2}$ is twice the active length $L_{1}$ of Mach-Zehnder interferometer hence the delay is $2\tau_{0}$ and the useful bandwidth is half of the maximal frequency $f\left(k=1\right)={\left(2\tau_{0}\right)}^{-1}$.

We have used the Michelson heterodyne configuration thus the beam in each arm is reflected before mixing the beat frequency represents the phase noise around the double of the modulator frequency $2\Delta f_{\mathrm{AOM}}$. Also the ambient environmental changes have influence on the optical fiber delay $2\tau_{0}$. The temperature change represents the relative length changes of $5\times 10^{-7}\;\;K^{-1}$. Air pressure changes around the fiber don’t have a direct effect and they are more difficult to detect. Thus, the typical fiber spool must be set into environmentally constant conditions blocking any ambient air changes [20]. Moreover any vibrational effects can be reduced by using the proper design and material for the spool support basis and passive and active anti-vibrational devices [26].

## 3. Experimental Set-up

### 3.1. Noise Suppression Scheme

**Figure 1:**
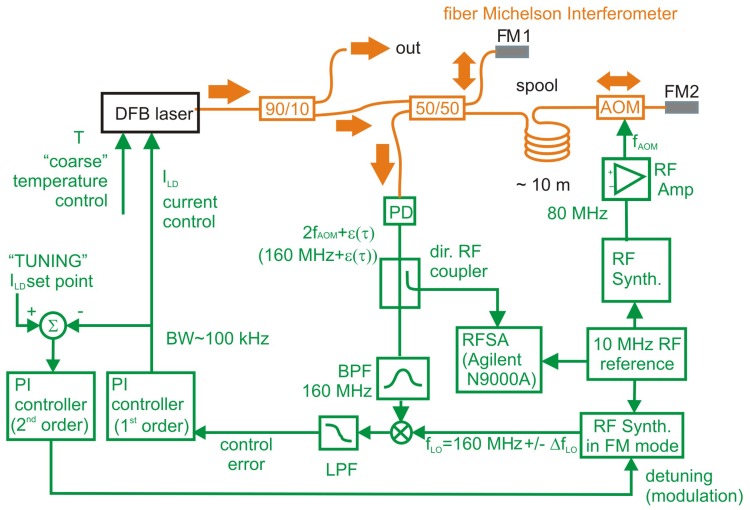
Schematics of the set-up of noise suppression of Distributed FeedBack (DFB) laser diode. 90/10 and 50/50 are fiber splitters, FM1 and FM2 are Faraday mirrors, AOM—acusto-optic modulator, PD—photodetector, DDS—direct digital synthesizer, BPF—band pass filter, RFSA—Radiofrequency Spectrum Analyzer, LPF—low pass filter.

In our experiment we have been using a Michelson unbalanced heterodyne interferometer. Schematics of the experimental set-up is in Figure 1.

The optical part (orange lines) consists of a Michelson unbalanced heterodyne interferometer (MUHI), photodetector and the laser. The DFB laser diode works at a central wavelength of 1542 nm with 1.5 MHz linewidth. Laser modulation bandwidth is 100 kHz and slow laser current tuning range is ±2 nm or 260 GHz. The light from the laser is split onto the 90/10 fiber splitter. 90% of the optical power is used as a stabilized output and 10% of the optical power enters the fiber Michelson interferometer. The core of unbalanced fiber Michelson interferometer consists of 50/50, 2 × 2 single mode fiber splitter. One arm of the interferometer was short with only roughly one meter of the single mode fiber ended by Faraday mirror 1 (FM1). The longer arm consists of a fiber spool represented by the single mode fiber of roughly 10 m, and an acousto-optic modulator (AOM) working at a fixed frequency of 80 MHz and Faraday mirror 2 (FM2). The 10 MHz reference signal is fed from the active H-maser. The complete set-up is put on a vibration isolated optical table and into a thermally isolated box. The AOM has been driven by a 80 MHz signal produced by a RF synthesizer. The optical frequency of the laser was shifted by AOM twice in the long arm. The beam from the long arm and from the short arm was mixed on the photodetector producing a RF beatnote with central frequency of 160 MHz containing the information about the laser phase noise in its sidebands.

The detection bandwidth and the sensitivity to phase fluctuations are determined by the delay $2\tau_{0}$ introduced by the beam travelling through the fiber spool in the long arm [22]. The RF signal from the photodetector is coupled into the RF spectral analyzer (RFSA) Agilent N9000A and analyzed. The spectrum of the signal is clipped by a 160 MHz band-pass filter. The resulting signal is translated to 0 Hz by a RF mixer. After low-pass filtering we get the control error signal which is fed into a P-I controller steering the optical frequency of the DFB laser by tuning the pump current $I_{\mathrm{LD}}$. The theoretical bandwidth of this controller is approx. 100 kHz. This type of noise suppression setup is sufficient if the length of all optical fibers was perfectly stable (with no thermal dilatations etc.) and if tuning of the optical frequency of the laser is not required. Otherwise we need to be able to control the DC set point of the DFB pump current. For this reason a "slow" 2nd order P-I controller is connected to the output of the "fast" 1st order P-I controller. The 2nd order servo loop slightly detunes the mixer local frequency $f_{\mathrm{LO}}$ from its nominal value of 160 MHz by the value $\Delta f_{\mathrm{LO}}$ of up to ±2 Hz. In a closed loop regime this detuning causes the noise suppressed laser to detune its optical frequency by $\Delta f_{\mathrm{LO}}/\tau_{0}\;\left(\mathrm{Hz}\cdot s^{-1}\right)$ [20]. As a result of combining these two servo loops we get a noise suppressed DFB laser that is kept at a desired operating point by keeping DC value of the pump current at a preset value. While keeping the fast servo in closed loop operation and by changing the set point of the second-order control loop, the central optical frequency of the laser changes so the whole system finds a new equilibrium state at the desired pump current value.

### 3.2. Displacement Measurement Set-up

**Figure 2:**
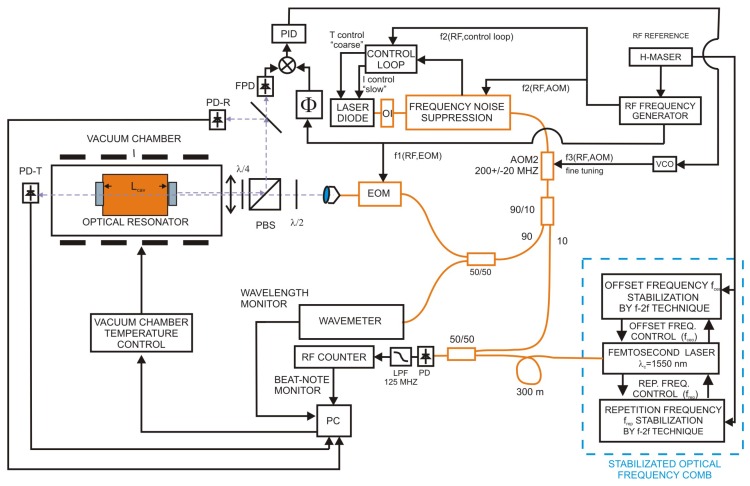
Scheme of the set-up for measurement of displacement of the Fabry-Perot cavity with Zerodur spacer under the temperature stable condition. EOM-electro-optic modulator, AOM2-frequency shifting acousto-optic modulator, VCO-voltage-control oscillator, OI-optic isolator, FPD-fast photodetector, PD-R-refection signal photodetector, PD-T-transmission signal photodetector, PBS-polarization beam splitter, PD-photodetector, LPF-low pass filter.

The displacement of the FPC [4] was measured in the temperature stabilized vacuum chamber on the vibration isolation table. Scheme of the set-up is in Figure 2. The cavity was made from a spacer of Zerodur glass ceramics in a confocal configuration. Two dielectric mirrors with a finesse of 339, deposited on 10 mm thick fused silica substrates were optically connected to the Zerodur spacer of 187.5 mm length, inner diameter of 12.7 mm and squared outer cross section of 40 mm × 40 mm. Both mirrors were anti-reflection coated. The FPC was placed on a four point holder in the axis of the temperature stabilized stainless steel vacuum chamber. The refractive index of air *n* in Equations (3), (5) and (6) has been reduced by keeping the FPC into the vacuum. The vacuum chamber was evacuated by a turbo-molecular pump and the vacuum was kept by an ion pump down to $10^{-5}$ Pa to avoid the vibrational effects. Temperature stabilization of the vacuum chamber was achieved by two resistance wires surrounding the chamber and controlled down to 0.5 K. The temperature of the Zerodur spacer was measured by two sensors on the FPC wall. The temperature at the FPC surface was stable down to 0.01 K over a period of tens of minutes.

The tunable distributed feedback (DFB) laser diode operates at a central wavelength of 1542.14 nm (194.40 THz) and can be tuned by the operating temperature control over more than 2 nm (250 GHz). The noise of the DFB laser was stabilized by frequency noise suppression in the unbalanced Michelson interferometer (see Section 3.1) using AOM fed by the RF signal from the RF frequency generator. The signal from the interferometer is used to control the current and in the long term by the temperature of the DFB laser diode. We were using traditional Pound-Drever-Hall technique (PDH) [8] to lock the laser to the FPC.

This method requires phase modulation of the initial DFB laser beam. This was done on an electro-optic modulator (EOM) at RF frequency of 20 MHz generated on frequency generator (f1(RF,EOM)). The fixed focal length collimator produced the collimated beam. The linear polarization was chosen by a half-wave plate λ/2 and a quarter-wave plate λ/4 that produces the rotation. The transmitted signal is detected on photodetector PD-T. Unlike the other typical set-ups [5] we were successful with one mode-matching lens [1] of focal length of 200 mm set in front of the vacuum chamber to match the cavity modes. The reflected signal was split by the polarization beam splitter (PBS) and one half of the reflected signal was detected by photodetector PD-R and the second half on fast photodetector (FPD) used to lock the DFB laser to the cavity. The error signal sent to PID controller was mix from the signal on the FPD and phase controlled RF signal f1(RF,EOM)) from the RF generator. The PID controller controlled the frequency of a voltage controlled oscillator (VCO) driving the second fiber acousto-optic modulator (AOM2) at (200±20) MHz.

The Er:doped stabilized femtosecond mode-locked laser working at a central wavelength of 1560 nm with repetition frequency 250 MHz worked as a reference optical frequency ruler and the optical frequency of the tunable DFB laser was counted via the beat frequency towards the comb teeth. The offset frequency of the mode-locked laser was generated and stabilized by standard f-2f interferometry [10] fully coupled in the fiber. Both the offset and repetition frequencies were stabilized to the H-maser.

The low power (<1 mW) output of the stabilized femtosecond laser comb in the spectral region 1500–1600 nm was delivered to the 50/50 splitter and mixed with 10% of the DFB laser diode shifted by the AOM2. The mixed signal was detected on the photodetector (PD) and low-pass filtered RF signal was counted on RF counter. In the same time, 50% of the light coming to the FPC was split and measured on wavelength meter Toptica, Ultimate wavemeter with 30 MHz resolution. This way we were monitoring the beat signal between the single mode DFB laser to the closest comb tooth while we have had information about the wavelength. The resolution of the wavemeter gave us enough precision to tell what comb line was the closest and contributes to the beat frequency.

All signals are monitored via a multi-channel system using the CAN-BUS system [24] and recorded on the computer (PC). The temperature inside the vacuum chamber was stabilized by a heating resistance belt covering the vacuum chamber.

## 4. Results

The single mode DFB laser diode was locked to three different modes of the Zerodur Fabry-Perot cavity. These three modes correspond to over 127 GHz tuning range of the DFB laser diode. Measurements of the uncertainty and the noise of the DFB laser diode locked to the cavity have been made for three different operating temperatures covering the whole tuning range of the laser.

### 4.1. Laser Frequency Noise Analysis

**Figure 3:**
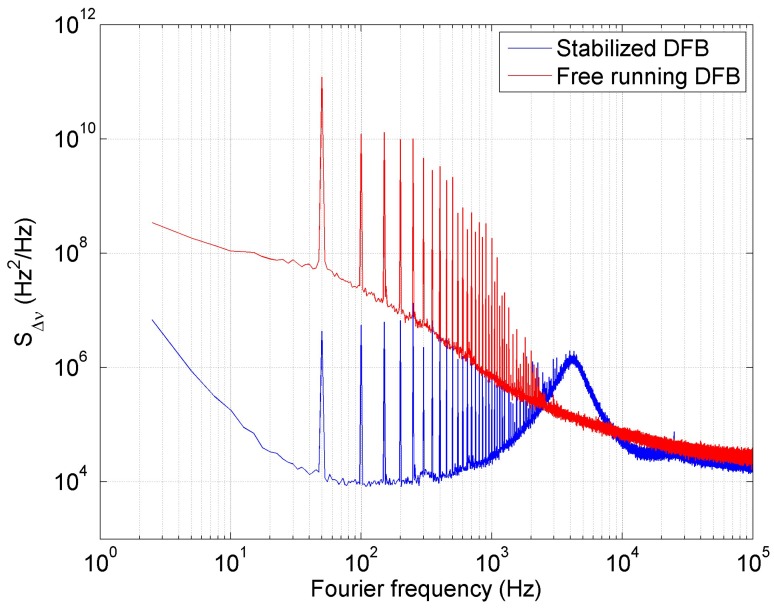
In-loop power spectral density of the frequency noise calculated from the RF spectrum measured on RF spectral analyzer Agilent N9000A (see RFSA in Figure 1).

Figure 3 represents the frequency noise analysis of the free running DFB laser diode (blue line) and the laser with frequency noise suppression (red line). The data in Figure 3 is based on the calculation from the data acquired on RF spectrum analyzer (RFSA) in Figure 1. The power spectral density of the in-loop beat frequency signal measured in dBm units by the RFSA has been normalized to the amplitude of the carrier and recalculated to the dBc units. Finally based on the assumption that for small variations of the frequency δν, the intensity variations of the interference signal δi depends linearly on the time delay of the interferometer $\tau_{0}$ and on the maximal intensity variations $\delta i_{0}$ [27]:

(12) $$\frac{\delta i}{\delta \nu }=2\pi \tau_{0}\delta i_{0}$$

the power spectral density data was recalculated to the power spectral density of the frequency noise [22].

The measurement was done under typical laboratory condition at $22.23^{\circ }$C with the lowest optical frequency of DFB laser diode. The free running and noise suppressed DFB laser diode noise was analyzed as well as the laser linewidth on the beat frequency measurement between the laser and the stabilized optical frequency comb as a reference.

The DFB laser diode frequency was controlled by diode current $I_{\mathrm{LD}}$ via the error signal to PI controller in the servo loop. As can be seen in the Figure 3 the noise level in the range from 3 Hz to 300 Hz decreased by more than 40 dB and by more than 20 dB in the frequency range from 300 Hz to 20 kHz.

In both cases we observed the 50 Hz noise peaks from the power supply and its harmonics. The 50 Hz electrical noise can be removed by supplying of the laser by the battery. The aim was to prove that the method worked even with the noise from the power supply. The laser stabilized in the servo control loop via interferometer in comparison to the free running DFB laser is represented by the noise level decrease and the noise peaks level is decreased as well. The control servo loop is limited by the electronics controlling the current and temperature of DFB laser diode. For the frequency around the 4 kHz the frequency and the phase noise of the locked signal exceeds the frequency and phase noise of the signal in the unlocked case. This additional noise is typical for feedback loops, known as "servo bumps". These servo bumps appear when the phase delay of the feedback signal exceeds *π* and the feedback changes from negative feedback to positive feedback. For that reason, the frequency noise for this Fourier frequency is even amplified instead of attenuated [28]. One can also observe important noise above so called *β*-separation line [29] thus we fit the linewidth of the laser by the Gaussian.

### 4.2. Frequency Beat Measurement

**Figure 4:**
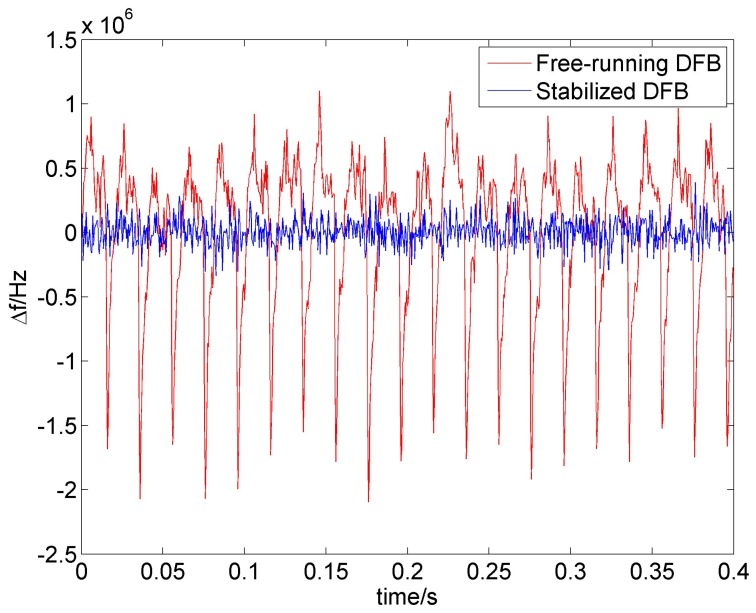
Temporal evolution of the beat frequency between the stabilized optical frequency comb and free-running DFB laser (red) and between the stabilized optical frequency comb and the noise suppressed DFB laser (blue).

The temporal evolution of the beat frequency is shown in the Figure 4. The data shows that the free-running DFB laser has noise with peaks around $0.5\times 10^{6}-1\times 10^{6}$ Hz as well as sharp peaks that change the optical frequency by $2\times 10^{6}$ Hz with the frequency of the 50 Hz representing the noise from the electrical. Applying the noise suppression method leads to a decrease of the noise by one order of magnitude with a small fluctuation of 50 Hz period.

The beat frequency in the temporal evolution have shown asymmetry that is projected to the beat frequency measurement. The linewidth has been analyzed by fitting favorably with a Gaussian profile.

We were able to decrease the linewidth from 1.5 MHz to 300 kHz increasing the symmetry of the laser linewidth. The DFB laser is emitting at a wavelength of 1542 nm with 2 nm tuning range. We measured the beat signal between the optical frequency comb and the DFB laser diode at different temperatures of the DFB laser corresponding to the highest, lowest and middle frequency in the tuning range of the DFB laser diode. The list of measured optical frequencies is in the Table 1. It represents the three measurement points within the continuous tuning range of the laser diode over (127760±40) MHz.

The error budget analysis based on the Equation (5) provides information about the uncertainty of the measurement. The estimation of the influence of the refractive index of residual gas under the vacuum of $10^{-5}$ Pa should be below $10^{-14}$ [30]. The measurement of the wavelength and the optical frequency by wavemeter has 30 MHz accuracy. This is enough precision to find *i* in Equation (1) because the spacing of the optical frequency comb or the repetition frequency is $f_{r}=250$ MHz. Analogically for the value *m* (Equations (2) and (5)) representing the number of the modes of the cavity that we are locking at (i.e., the FSR)-$\Delta \nu_{\mathrm{FSR}}=400$ MHz. Limiting factor for the uncertainty of the measurement is the stability of the optical frequency comb reference, i.e., the repetition $f_{r}$ and the offset frequency $f_{0}$. We have already shown the stabilization technique of the optical frequency and the uncertainty of the measurement of the wavelength goes down below 400 Hz [24].

The beat note frequency between the noise suppressed DFB laser diode and the closest optical frequency comb has been measured on three different measurement points: No. 1–3 (see Table 1) corresponding to three different temperatures of DFB diode. We have proven that with lower than 300 kHz resolution represented by the linewidth of the frequency suppressed laser diode and uncertainty represented by the reference based on optical frequency comb close to 300 kHz (see reference [24]) we are able to continuously track the cavity displacement over the range of 128 GHz. This corresponds to $319\times \Delta \nu_{\;\mathrm{FSR}}$ or 0.246259 mm with the relative resolution $1.5\times 10^{-9},$ that for a 0.178 m long cavity corresponds to 0.3 nm (sub-nm) resolution. The length precision is limited by the reference in the optical domain that corresponds to the optical frequency comb stabilized to H-maser thus it can go down to 3 pm.

## 5. Conclusion

We have measured and proven the frequency noise suppression on a DFB laser diode. The frequency noise level was suppressed by more than 40 dB for Fourier frequencies of 10–300 Hz and by more than 20 dB for Fourier frequencies of 300–1000 Hz. The measurement of the linewidth of the free-running and stabilized DFB laser diode showed the reduction of the linewidth from 1.5 MHz to 300 kHz. The noise suppressed DFB laser diode was used in the set-up for measurement of displacement of a 0.178 m long Fabry-Perot cavity and we have shown a relative resolution of $1.5\times 10^{-9}$ that corresponds to 0.3 nm (sub-nm) for theoretical displacement of 0.25 mm. 

## Figures and Tables

**Table 1:** List of measured data points (t-diode case temperature).

No.	t (°C)	Opt. Frequency (MHz)
1	22.230±0.001	19454984(5)±30
2	14.993±0.001	19462089(6)±30
3	8.695±0.001	19467760(6)±30
